# Using mobile brain/body imaging to advance research in arts, health, and related therapeutics

**DOI:** 10.1111/ejn.15313

**Published:** 2021-06-07

**Authors:** Juliet L. King, Francisco J. Parada

**Affiliations:** ^1^ Department of Art Therapy The George Washington University Washington DC USA; ^2^ Department of Neurology Indiana University School of Medicine Indianapolis Indiana USA; ^3^ Centro de Estudios en Neurociencia Humana y Neuropsicología. Facultad de Psicología Universidad Diego Portales Santiago Chile

**Keywords:** 4E cognition, art Therapy, creative arts therapies, mobile Brain/Body Imaging, neuroaesthetics

## Abstract

The uses of mobile brain/body imaging (MoBI) are expanding and allow for more direct study of the neurophysiological signals associated with behavior in psychotherapeutic encounters. Neuroaesthetics is concerned with the cognitive and neural basis of art appreciation, and scientific correlations are being made in the field that might help to clarify theories claimed in the creative arts therapies. Yet, most neuroaesthetics studies are confined to the laboratory and do not propose a translation for research methods and clinical applications. The creative arts therapies have a long history of clinical success with various patient populations and will benefit from increased scientific explanation to support intervention strategies. Examining the brain dynamics and motor behaviors that are associated with the higher complex processes involved in artistic expression offers MoBI as a promising instrumentation to move forward in linking ideas from neuroaesthetics to the creative arts therapies. Tracking brain dynamics in association with behavioral change allows for more objective and quantitative physiological monitors to evaluate, and together with subjective patient reports provides insight into the psychological mechanisms of change in treatment. We outline a framework that shows how MoBI can be used to study the effectiveness of creative arts therapy interventions motivated by the 4E approach to cognition with a focus on visual art therapy. The article illuminates how a new partnership among the fields of art therapy, neuroscience, and neuroaesthetics might work together within the 4E/MoBI framework in efforts to advance transdisciplinary research for clinical health populations.

## Graphical abstract

The article illuminates how a new partnership between the fields of art therapy, neuroscience, and neuroaesthetics might work together within the 4E/MoBI framework in efforts to advance transdisciplinary research for clinical health populations.

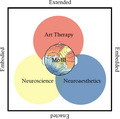

## 1 INTRODUCTION

The original development of mobile brain/body imaging (MoBI) at the Swartz Center for Computational Neuroscience at the University of California, San Diego (Makeig et al., 2009), expanded the ability to measure brain/body dynamics in real‐world environments; a current scientific challenge (Ladouce et al., 2017; Matusz et al., 2019; Parada & Rossi, 2020; Zaki & Ochsner, 2009). Doing so offers valuable insights for cognitive neuroscience (Gramann et al., 2014; Ladouce et al., 2017; Shamay‐Tsoory & Mendelsohn, 2019) and neurorehabilitation (Petrini et al., 2019; Spychala et al., 2019). Furthermore, reaching into neuroaesthetics (Cruz‐Garza et al., 2019; Djebbara, Fich, & Gramann, 2019) and architecture (Djebbara, Fich, Petrini, et al., 2019). Overcoming restrictions of traditional brain imaging technologies with MoBI allows studying cognition in natural settings and provides an ecologically relevant approach to the connections among the brain, body, and behavior (Gramann et al., 2011, 2014; Ladouce et al., 2017). The use of MoBI to study clinical health populations is apt for expanding and allows for more direct study of the neurophysiological signals associated with behavior in psychotherapeutic encounters. Here, we will outline a framework that shows how MoBI can be used to study the effectiveness of creative arts therapies interventions (i.e., visual art, music, dance movement, and psychodrama) with a focus on visual art therapy. The framework is motivated by the recent embodied, extended, embedded, and enactive approach to cognition (known as the 4E approach to cognition as in Newen et al., 2018; Parada & Rossi, 2020), and will help integrate neurosciences, arts, and related therapeutics. We propose MoBI as a viable tool to advance our understanding of how best to improve clinical health outcomes in patient populations.

The MoBI community opened new doors with the 2016 International Conference on Mobile Brain‐Body Imaging and the Neuroscience of Art, Innovation and Creativity and continues to expand (Cruz‐Garza et al., 2019; Gramann, 2018; Gramann et al., 2017; King, 2018). Here, partners in the sciences, arts, and therapeutics discussed the use of MoBI to foster knowledge in their respective fields. The arts may provide universal truths in a graspable form (Guyer, 2016), while artistic expression can bring to awareness less conscious psychological content (Seth, 2019). Understanding the cognitive and emotional components of aesthetic productions can be enhanced and grounded through the concreteness afforded through (neuro)physiological measures accompanied by behavioral observations and subjective associations from the artist and art therapist. Comparing (neuro)physiological signals with underlying behavioral states leads to greater understanding of physical and mental processes (Brouwer et al., 2015; Krakauer et al., 2017). Different natures and levels of organization that characterize psychological and physiological phenomena present both an epistemological and methodological problem (King, 2018). Hence, theory‐driven research along with collaborative, translational, and transdisciplinary approaches are needed. Artistic expression—heavily relying on movement—involves an engagement of visual, perceptual, sensory, cognitive, and emotional systems. Furthermore, people with limited mobility experience the beneficial effects of making and viewing art (King & Pascuzzi, 2018; Scott et al., 2019).

Neuroaesthetics and art therapy have experienced some of the same methodological challenges, although the goals and purpose of inquiry are different. Neuroaesthetics and art therapy help to explain that human behavior and cognition represent inherent fundamental collections of highest levels of cerebral function (King, 2016). Scientific correlations that are being made in neuroaesthetics might be used to help explore and clarify theories claimed in art therapy. Neuroaesthetics is concerned with the cognitive and neural basis of art appreciation and production. Hence, this field of inquiry ought to be relevant for art therapy. Still, most neuroaesthetics studies are confined to the laboratory and do not examine changes as they are happening in the wild. Art therapy encounters are an example of an ecological setting in which the nature of dynamic change is most important. Following the main principles of the 4E approach, cognition is an emergent product of the body's diachronic interaction with the world in a dynamically changing environment (Varela et al., 1991; Wilson, 2002). Here, MoBI scaffolds the study of both cognitive and emotional states (Parada & Rossi, 2020). Such studies advance the field of neuroaesthetics, art therapy, and related professions. The present article intends to brighten beginning stages for how art therapy, neuroscience, and neuroaesthetics might work together following the 4E/MoBI framework as the means to advance transdisciplinary research for clinical health populations (Dikker et al., 2020; Parada & Rossi, 2020).

### 1.1 Neuroaesthetics: A promising partner to psychotherapy

Neuroaesthetics is an emerging subdiscipline of cognitive neuroscience investigating biological mechanisms involved in making and viewing art (Chatterjee & Vartanian, 2014; Skov & Vartanian, 2009). Neuroaesthetics studies help in identifying and explaining interactions of visual information processing systems in aesthetic experiences. Zeki (1999) coined the term several decades ago, claiming that art and physiology are intertwined. Chatterjee (2014) asserts that while neuroaesthetics helps in bridging empirical aesthetics with cognitive neuroscience, the inquiry involves interactions between the observer and the artwork itself. This concept raises ideas for how empirical evidence that typically is established in the laboratory can be translated to study intra‐ and interpersonal human real‐world interactions. For example, neuroaesthetics findings suggest that the aesthetic experience of visual artwork is characterized by the activation of sensorimotor areas, core emotional centers, and reward‐related centers (Di Dio & Gallese, 2009). These data might be used as a framework to test the neurobiology of motivation and mood in a clinical context. Further, aesthetic evaluation of the arts activates the same neural systems as primary reinforcers such as food and drink (Chatterjee & Vartanian, 2016). These data offer opportunities to consider how neuroaesthetics studies may contribute to the understanding of complex psychological constructs such as reward and motivation and their implications to mental health (Strauss et al., 2014). For example, it has been found that art making engages the reward system and activates dopamine production (Lambert, 2008), which influences the psychological benefits of art making in the therapeutic context (Kaimal et al., 2017; Kaimal, 2019). Translating data generated from experiments in neuroaesthetics will help to develop novel hypotheses to test these activation patterns in dynamic therapeutic settings and might offer insights for how to further define and characterize phenomenological elements of human relatedness. These are more difficult to study and comprehend yet integral to the psychotherapeutic process.

Experience is a phenomenological endeavor and one that does not precisely fit into traditional scientific methodology (Varela, 1996). The scientific study of neuroaesthetics has faced challenges due to the subjective nature of aesthetic experiences, metaphorically conceptualized as a snowflake: “On the whole, it is similar to any other; in the details, it is unique, ephemeral, and unrepeatable” (Pearce et al., 2016, p. 272). Historically, studies in neuroaesthetics focus on the viewer’s engagement with the aesthetic experience. Tinio (2013) bridges making and viewing art while accounting for the individual differences in subjective experience in his *Mirror Model of Art*. This model describes commonalities and distinctions between art making and viewing, linking the fields of aesthetics and creativity, where the interactions between the making and observing of art are most salient (Tinio, 2013). His model emphasizes that the experience of artwork has purpose beyond what it looks like and how it is classified, in that *how* and *why* it was created holds just as much meaning (p. 274). Considering the convergence of observation and production of artwork probes insight into human interaction with artistic materials, the processes of making art, and observations of the art once it is created. Making art and engaging in dialogue surrounding its creation help to objectify personal experiences and perceptions (Wadeson, 2010). Tinio’s model could provide a framework for research protocols that expand the knowledge of *how* and *why* the arts may be engaged to understand both health and disease states (King, 2016). By studying the links among artistic production, neural deficits, symptoms, and consequent behaviors, knowledge gaps could be identified, and cogent recommendations made to translate neuroaesthetics data for use in research with clinical health populations.

Even though neuroaesthetics does not necessarily focus on psychotherapeutic encounters, the methods used to understand how the brain is related to artistic experiences could extend to therapy (Chatterjee, 2011). While studies in neuroaesthetics hold implications for therapeutics, a direct proposition has not yet been made to the best of our awareness. Park et al. (2018) investigated neural activity in people with autism spectrum disorder (ASD) compared to a non‐clinical population during a task that compared two types of artwork, landscapes and fractal patterns. The study reports that brain activation patterns associated with aesthetic experiences in ASD may differ from those without and point to considerable implications for social rehabilitation, designing educational curricula, and establishing art therapy processes for people with ASD. This is consistent with the high heterogeneity of ASD, which could map into the diverse neurodynamics reported in the literature (Kovarski et al., 2019; Latinus et al., 2019).

A clinically important example for the translational role of MoBI to explore the intersection of neuroscience, art therapy, and neuroaesthetics through the understanding of brain dynamics and symptom presentation is that of Parkinson’s disease. There is a growing and substantial body of work that explores the artistic perceptions of people who have Parkinson’s disease and proposes neurobiological links that offer insight into treatment potentials (Lauring, Ishizu, et al., 2019; Lauring, Pelowski, et al., 2019; Pelowski et al., 2020). An increase in artistic ability and awakening of creative potential in patients with Parkinson’s disease has been shown despite impairments in visual spatial processing and motor control (Lauring et al., 2019). Exploring the neural basis of aesthetic perception offers opportunity to understand complex cognitive, motor, and sensory functions and their connection to human behavior. For the clinical researcher or physician focused on Parkinson’s disease, art making or viewing, combined with changes from the disease, represents intriguing potential for early diagnosis, rehabilitative therapy (Haaxma et al., 2015; Lim et al., 2009), and for the understanding of non‐motor symptoms (Chaudhuri et al., 2015; Martinez‐Martin et al., 2011).

Welcoming MoBI to this discussion is indicated. Studies using qualitative electroencephalogram (qEEG) to date are usually based on recordings from stationary patients unless the equipment used allows for mobile tracking. Patients often experience freezing when trying to walk, their limbs can fail abruptly (e.g., when walking into a narrow space, such as a doorway), and their environment clearly affects their symptoms and abilities to walk. Patients have trouble initiating walking but if there is a visual line placed in front of them, they can step over it and begin walking. In other words, the simple environmental modification affords the person a better perception–action fitting. Furthermore, pointing to the direct relationship between the environment and control of motor function. The use of MoBI would enable—for the first time—an evaluation of cortical physiology in patients who have Parkinson’s disease in their states of greatest symptomatology (i.e., mobility). Cucca et al. (2018; Cucca et al., 2021) have propelled this direction by exploring the neural connections of visual spatial processing and artistic experiences with people who have Parkinson’s disease in efforts to assess art therapy as a valuable neurorehabilitation intervention. Anxiety, fear, and stress are prevalent non‐motor symptoms of Parkinson’s disease that can aggravate tremor in Parkinson’s disease as in other involuntary movement disorders (such as dystonia and essential tremor) (Dissanayaka et al., 2010). In many cases, the environment is a source of enhanced stress and anxiety, be it from fear of falling, fear of freezing, or more complex thoughts of function and perception. Importantly, the brain autonomously and continuously senses the body (i.e., interoception). MoBI, allowing the acquisition of brain/body physiological signals during a natural therapeutic encounter, opens the door for studying *interoception in the wild*. Quantifying visceral signals linked to anxiety in a scalable fashion—from laboratory to less‐structured naturalistic settings—will allow therapists and researchers to better identify successful, personalized interventions, and strategies (Quadt et al., 2018). For example, coupling a felt sensation of subjective anxiety with a heartbeat tracking task and a behavioral measure could provide insights into a person’s ability to consciously perceive their bodily signals, which might be understood as interoceptive accuracy (Quadt et al., 2018). Furthermore, consciously connecting subjective physiological sensations and behavioral states in patients with Parkinson’s disease is often neglected and indicates a need to study the mechanisms involved with environmental and experiential processes and the loss of optimal motor function, with the goal of using such data to create enhanced therapeutic interventions, including those involved with art therapy and aesthetics.

Neuroaesthetics is contributing to studies that explore the impact of the environment on perception (Djebbara, Fich, & Gramann, 2019; Djebbara, Fich, Petrini, et al., 2019). For example, Vartanian et al. (2013) studied how architecture influences the dynamic of approach–avoidance. This is when the human drive to pursue reward and avoid harm are incompatible and highly variable from person to person, yet follows a premise that humans are motivated to approach things that are sustaining and avoid things that are threatening (Zorowitz et al., 2019). While a growing area of inquiry, studies that combine elements of neuroaesthetics and architecture might be used as a theoretical framework to further understand the role of the environment as a healing agent and therapeutic tool.

Neuroaesthetics has generated momentum through experimental research, yet the field might be considered transdisciplinary by design in that it invites elements of artistic creation to the realm of cognitive neurosciences (King, 2018). Traditions of research in empirical aesthetics and cognitive neuroscience have helped to clarify the scope of the profession and provide a context for investigating the complex brain regions involved in aesthetic experiences that might be applied in a way that is also complementary to approaches in the humanities (Pearce et al., 2016). Marin (2015) emphasizes how interdisciplinarity research in neuroaesthetics in which arts, humanities, and the sciences collaboration would promote a broader understanding of aesthetic experiences. We agree with this stance and see the inclusion of the creative arts therapies would uniquely contribute to understanding aesthetics and its applications to human interactions that take place in psychotherapy.

The complex conditions that contribute to the dynamic changes in psychotherapy are intricate, complex, and difficult to study (Koole et al., 2020; Parada & Rossi, 2020). Simplifying and reducing concepts is the goal, as scientists strive for ways of knowing that are clear and replicable (Poeppel & Adolfi, 2020). Paraphrasing Einstein, “Everything should be made as simply as possible, but no simpler” (in Funnell & Rogers, 2011). Psychotherapy is a natural, semi‐structured interactional setting that reduces problematic symptoms and improves well‐being (Cozolino, 2017). A core foundation of psychotherapy is the human relationship that emerges between patient and therapist. An attuned therapeutic relationship helps to effect psychological change in the patient, leading to self‐awareness, and—eventually—a reduction in clinical symptoms. Simultaneously, the most important and the most difficult to study, the therapeutic relationship (Colli et al., 2019; King‐Casas et al., 2008; Ryu et al., 2020; Talia et al., 2017), may be explored with more clarity with the introduction of neuroaesthetics methods.

Learning more about the areas of the brain activated in social engagement is crucial to understanding the therapeutic relationship. Emerging research in neuroscience has shown that people who have closer relationships also share neural networks, largely in part due to the functioning of the default mode network (DMN), which allows our brains to shape and be shaped by another (Yeshurun, Nguyen, & Hasson, 2021). Here, neuroaesthetics might offer a framework to study the implications of the therapeutic relationship by clarifying functionality in the DMN associated with aesthetic experiences. For example, Belfi et al. (2019) suggest that structures such as the DMN help to track the internal state of the individual during aesthetic experiences by explaining the relationship between top‐down and bottom‐up sensory processing that contributes to increased engagement with the visual input. Vessel et al. (2012) found that the DMN is activated during highly aesthetic experiences that the viewer perceived as intense. These findings illuminate the cognitive processes involved in aesthetic experiences and could provide insights into self‐perception. Furthermore, it could be said that a person’s taste in art is connected to their identity (Kandel, 2016, p. 184). Bolwerk et al. (2014) linked neural effects of visual art production with psychological resilience through the investigation of the DMN with functional magnetic resonance imaging (fMRI). Translating scientific data to understand therapeutic implications with clinical health populations could be made more possible when applying the rigorous methods of cognitive neuroscience and neuroaesthetics to research in art therapy.

The field of art therapy is expanding its theoretical understanding of the functional networks involved in artistic engagement (Lusebrink & Hinz, 2020) and has begun to investigate changes in the DMN secondary to specific intervention strategies with traumatized populations (Walker et al., 2018). The partnership of neuroaesthetics and art therapy provides opportunity to study challenging psychological domains such as engagement and motivation, self‐awareness and agency, identity and resilience, all of which are integral to the therapeutic healing process. MoBI will provide an insight into the brain/body physiological dynamics underlying first‐person experience. Body location in a natural environment and the corresponding visceral states are foundational aspects of subjective perception (Blanke & Metzinger, 2009), which will further afford the sense of body ownership and, ultimately, the configuration of agency and the self (Azzalini et al., 2019).

Creative arts therapies (i.e., visual art, music, dance movement, and psychodrama) are a promising partner to neuroaesthetics. Creative arts therapists have long understood that thoughts, emotions, and urges for action are extended throughout therapy and that artistic expression within the context of the therapeutic relationship helps to concretize these domains. Trained also as “talk therapists” (i.e., traditional psychotherapists), creative arts therapists are masters‐level clinicians who specialize in understanding how to use the creative process and its products to explore the underlying psychological mechanisms of change in therapy. Creative arts therapies have produced substantial scholarship documenting their clinical value and see that the creative–expressive process “engages physiological sensations, emotions, and cognition. Furthermore, facilitating verbal and non‐verbal symbolization, narration, and expression of conscious or unconscious conflict and sense‐making through internal and external dialogue and communication between oneself and others” (Shafir et al., 2020, p. 1).

However, research remains challenging when multiple psychological processes are involved in therapy and is made even more complex with the additional components of artistic expression (King & Kaimal, 2019). Understanding multidimensional and complex systems that are engaged when a person is involved in aesthetic experiences could offer insights into how to combine neuroaesthetics and creative arts therapies (although see Matusz et al., 2019; Parada, 2018; Parada & Rossi, 2017). Here, MoBI might be critical for this translation, in that artistic expression—heavily relying on movement—involves an engagement of visual, perceptual, sensory, cognitive, and emotional systems. For brevity, we will focus on visual art therapy, although what we propose may serve as foundation for inquiry across all of the creative arts therapies, along with research at the intersection of arts, health, and well‐being.

### 1.2 Art therapy: An established profession

The arts have been used to heal since the beginnings of human culture. This healing process involves physical engagement in the materials, processes, and products of their creation (McNiff, 1992). Engagement with the arts either as an observer or creator can modulate mood, emotion, and psychological states (Stuckey & Nobel, 2010). Recent evidence suggests the possibility of exploring the characteristics of sense making and the overall human experience during therapeutic encounters (King, 2018; Parada & Rossi, 2020). These insights promote the value of the arts in the healing process and make it possible to create more effective therapeutic strategies for clinical populations (King & Kaimal, 2019).

The American Art Therapy Association (AATA, http://www.arttherapy.org/) defines art therapy as an integrative mental health and human services profession that enriches the lives of individuals, families, and communities through active art making, creative process, applied psychological theory, and human experience within a psychotherapeutic relationship. Art therapy in the USA was established in the mid‐20th century. Its source lies in the natural world, where cave paintings, fertility figures, sand mandalas, and ritual masks are used to symbolize the human experience and represent the historical antecedents of modern art therapy (Rubin, 2009). Art therapy is a hybrid discipline based primarily on the fields of art and psychology (Vick, 2003) and the profession has strong roots in psychoanalysis and depth psychology. A particular emphasis is given to the value of creative, verbal, and non‐verbal symbolic self‐expression as a way to heal and as a tool for communication. In some ways similar to the evolution of neuroaesthetics, contemporary art therapy theory has been enhanced with strong influences from Gestalt principles and has benefitted from the consideration of psychobiological approaches (Leder et al., 2013). Art therapists inherently see art as isomorphic, holding a multidimensional perspective of the person who made it, and the process involved in its creation holds as much—if not more—meaning than the image itself.

Art therapy theories evolved over time and inform assessment and intervention strategies that ameliorate mental and medical health symptoms, foster resilience, and strengthen relationships. For example, Czamanski‐Cohen & Weihs (2016) emphasized the need to translate clinical knowledge that is unique to art therapy and proposed a *Bodymind model* that examines the mechanisms of art therapy and their effect on health. Kaimal (2019) proposed an evolutionary framework to advance research that is grounded in the human response to external threats that are subjectively experienced, processed on conscious and unconscious levels, and potentially influenced by art making and other creative activities. Outcomes studies have shown a body of quantifiable data to support the claim that art therapy is effective in treating a range of mental health disorders and accompanying symptoms and show art therapy was of key benefit to psychological and social recovery (Eaton et al., 2007; Slayton et al., 2010; Uttley et al., 2015; Van Lith et al., 2013). Randomized control trials have elicited significant data that showed decreased anxiety and increase in quality of life with children who have asthma (Beebe et al., 2010), patients with breast cancer (Jang et al., 2016; Monti et al., 2006), and a significant improvement of mood in non‐clinical populations (Babouchkina & Robbins, 2015; Bell & Robbins, 2007). Most theories of art therapy claim that artistic expression bypasses verbalization in externalizing internal processes, and assumes that it engages the sensorimotor system, recruits cognitive processes, and has the ability to balance emotional dysregulation through the re‐attunement of healthy attachment patterns (Chapman, 2014; King, 2016; King et al., 2019; Lusebrink, 2010; Malchiodi, 2015).

Neuroscience‐informed protocols have been applied when working with traumatized patients and have demonstrated that art therapy: (a) facilitates the organization and integration of traumatic memories; (b) reactivates positive emotions and serves as a vehicle for exposure and externalization of difficult content; (c) reduces heightened arousal responses; (d) enhances emotional self‐efficacy and maintains a space for the exploration of self‐perception and psychic integration; and (e) enhances the development of identity (Chapman et al., 2001; Hass‐Cohen & Carr, 2008; King, 2016; Malchiodi, 2020; McNamee, 2005, 2006; Tripp, 2007). However, the lack of scientific evidence to buttress these claims, perhaps stemming from a lack of clear research strategies, remains a challenge (Kubovy, 2019; Orkibi & Feniger‐Schaal, 2019).

The visionary work by Kagin and Lusebrink (1978) sought to merge neuroscience principles with art therapy approaches, in a framework called the expressive therapies continuum (ETC). This has been expanded and updated to provide a more thorough understanding of mind and brain functioning with reference to art‐making applications in the clinical context of art therapy (Lusebrink & Hinz, 2016, 2020). The ETC identifies how to approach therapeutic art interventions and describes how media properties may evoke emotional and cognitive responses when paired with thoughtful intervention strategies (Hinz, 2019). The ETC seeks to clarify the hierarchical nature of visual information processing and is theoretically aligned with brain function and activity along three levels: (a) the kinesthetic/sensory level (K/S): It has been proposed that artistic expression on the K/S level reflects brain activity in the primary sensory motor cortices that manage kinesthetic and sensory input and is presumed to be processed through the basal ganglia, primary motor cortex, and somatosensory cortex. From here information is forwarded to or integrated into other brain areas for further processing; (b) perceptual/affective level (P/A): Visual information processing on the P/A level appears to represent functions of the ventral visual stream, with the influence of the affective component of the P/A level representing activity of the anterior insula and the amygdala in the limbic cortex; and (c) cognitive/symbolic level (C/S): The C/S level engages large‐scale brain networks (LSBN) involved in processing cognition and adds insights into the cognitive processes operating during art therapy such as the central‐executive network, the salience network, and the default‐mode network. These levels are integrated through level: (d) creative expression along with the artists’ interactions with media and dialogue and with the image itself (Lusebrink, 2004, 2010; Lusebrink & Hinz, 2020). Similarities between aspects of the ETC and LSBN models along with the fluctuations observed in fMRI and artistic productions might be immature at this time but also inform interventions and research strategies moving forward (Lusebrink & Hinz, 2020).

The ETC gives an example for how neuroscience helps to identify the “active ingredients” of art therapy (Kapitan, 2012); and leads toward the identification of mechanisms that produce change through treatment and tests common assumptions that historically accompanied its applications in practice 1. It is hypothesized that art materials have distinct qualities and engage different areas and connectivity networks of the brain and this framework has been the foundation for scientific study that explores the brain activity underlying art making with different materials to illustrate the shifts theorized with the ETC. For example, Kruk et al. (2014) found that there were significant differences when comparing a drawing task to a clay sculpting task and that both conditions were significantly different from baseline. The study found that art making involves the right parietal lobe and includes a pattern of increased alpha wave activity which supports previous study (Belkofer et al., 2014). These results may indicate the potential for relaxation through creative opportunities generated by drawing tasks and the activation of memory processes, meditative states and spatiotemporal processing (King and Kaimal, 2019). This research also illustrated a frontoparietal network involved in art making that has been found in previous work (Belkofer & Konopka, 2008; Solso, 2000; Yuan & Brown, 2014) and provides ideas for how media properties might be tested further. These preliminary studies offer a theoretical foundation from which to test art therapy interventions in studies of efficacy and effectiveness.

An activation likelihood estimation (ALE) meta‐analysis (Griffith & Bingman, 2020) tested the ETC hypothesis (Lusebrink, 2010) that different levels of the ETC would be supported by different levels of functional brain activation. The results found that cognitive drawing (operationalized by internally cued drawing stimuli or objective drawing content) would be associated with activation of the prefrontal and cingulate cortices and perceptual drawing was associated with cerebellum, frontal, and parietal lobe function, including the motor and somatosensory cortices, and the dorsal visual pathway was engaged during both cognitive and perceptual drawing. Haiblum‐Itskovitch et al. (2018) considered heart rate variability (HRV) measured with electrocardiography (EKG) following engagement with different art materials (pencil, gouache, and oil pastels) and found that the materials brought about significant changes in emotional response along with HRV. Further, both sympathetic and parasympathetic nervous system responses occurred specifically with the use of oil pastels were related to emotional valence, and may point to emotional engagement. These findings move the ETC framework toward more scientific validation and serve as foundations for future research.

Our understanding of the physiological mechanisms by which art therapy exerts its effects is advancing (Belkofer et al., 2014; Czamanski‐Cohen & Weihs, 2016; Hass‐Cohen & Findlay, 2015; Kaimal et al., 2016, 2017; King et al., 2017; Lusebrink, 2010; Walker et al., 2018) and the field is beginning to more accurately identify the underlying physiological mechanisms associated with emotion, cognition, and learning that enhance the capacities to change through therapy (King et al., 2019). For example, Beerse, Van Lith, and Stanwood (2020) found that anxiety and perceived stress were reduced, and salivary cortisol outcomes were used to measure the differences between a mindfulness‐based art therapy (MBAT) intervention compared to an art‐making task in college‐aged students. This work has built upon other studies that have used physiological measures to show fluctuations in brain activity when engaging in different types of art‐making tasks. For example, Kaimal et al. (2017) found that activities including free drawing, doodling, and coloring activated reward pathways using functional near‐infrared spectroscopy (fNIRS) and fMRI; and Walker et al. (2018) found that improved thalamic activity was associated with imagery referencing community, purpose, and belonging in military service members with brain injury and post‐traumatic stress. Research using technologies such as electroencephalographic (EEG) (Belkofer et al., 2014; Belkofer & Konopka, 2008; King et al., 2017), heart rate variability monitoring (Haiblum‐Itskovitch et al., 2018), fNIRS (Kaimal et al., 2017), and fMRI (Walker et al., 2018) propel the inquiry of these principles with contemporary technologies. Clarifying the psychological mechanisms of art therapy is enhanced with technologies that provide insight into the mechanisms and neural processes involved in art therapy and help to quantify the benefits and dosage in defined patient populations.

Based on clinical observations and scientific theory, King (2016) developed a set of precise principles to motivate integration of art therapy and neurosciences: (a) the art‐making process and the artwork itself are integral components of treatment that help to understand and elicit verbal and non‐verbal communication in a therapeutic relationship; (b) creative expression is healing and life enhancing; and (c) the materials and methods used effect self‐expression, assist in emotional self‐regulation, and are applied in specialized ways. Conceptualizing research approaches within these principles helps to distill and organize components that contribute to mechanisms of change in art therapy and offer opportunities for research collaborations. For example, brain‐to‐brain synchrony as a way of understanding human interaction and engagement. The work of Dikker et al. (2017) contributes to understanding the first and most important principle, that of the therapeutic relationship, through recording brain‐to‐brain synchrony in social contexts (Bhattacharya, 2017; Parada & Rossi, 2017). The study found that in a classroom environment brain‐to‐brain group synchrony was able to predict classroom social dynamics and is driven by a combination of stimulus properties (i.e., teaching styles) and individual differences, including student preference and teacher likability. This information can be used as a foundation to explore the relationship that exists between therapist and patient, expanding the inquiry into the mirror neuron system and domains such as empathy and attachment patterns that are important factors that contribute to therapeutic success (Dikker et al., 2017). This also offers consideration for how MoBI person‐to‐person synchronization might even be used to assess the “attunement” of therapist and patient, inviting a wide and expansive realm of scientific study that can significantly increase the understanding of *how* and *why* psychotherapy works. Following advances in modern neuroscience, putting theory (Parada & Rossi, 2018; Varela et al., 1991), ecological behavior (Krakauer et al., 2017; Shapiro, 2019), and the search of mechanisms (Bechtel, 2012; Craver, 2007; Rojas‐Líbano & Parada, 2019) must be fundamental for art therapy research programs.

### 1.3 Mobile brain/body imaging implemented as a three‐level model for research in neuroscience, arts, and therapeutics

During the 1990s, theories from the first half of the 20th century (Berthoz, 2008; Gibson, 2014; Merleau‐Ponty, 1976) gave way to discussions emphasizing the dynamic relationship among mind, body, and environment (Andy & David, 1998; Flor & Hutchins, 1991). Cognition is conceived as a phenomenon rooted in biology, actively and dynamically coupled with the outside world as it extends into both physical and sociocultural structures and embeds with its ecological niche (Parada & Rossi, 2018, 2020). This perspective has been recently “unified” within a heterogenous and pluralistic *Embodied*, *Extended*, *Embedded*, and *Enactive approach to cognition* 2 (Newen et al., 2018). Discussing the implications of this approach is beyond the scope of the present piece (for a global and deeper treatment of the topic, see the special edition by Gonzalez‐Grandón & Froese, 2018; the special edition by Menary, 2010; the book edited by Newen et al., 2018; and the MoBI‐relevant piece by Parada & Rossi, 2020, in the present special edition). Nevertheless, three main tenets of the 4E approach are as follows: (a) cognition is a product of cerebral and extra‐cranial dynamics and processes (Varela et al., 1991), (b) cognition is a product of an agent’s interaction with others in the world (De Jaegher et al., 2010; Rojas‐Líbano & Parada, 2019), and (c) cognition is a product of brain/body morphology operating in the world (Parada & Rossi, 2018; Sporns, 2010; Varela et al., 1991). The intersection between arts and therapeutics can be a paradigmatic example of what Gramann et al. (2014) called *natural cognition*. Hence, measuring neurophysiological dynamics during art therapeutic interactions is a methodological challenge that embraces the partnership of neuroaesthetics and art therapy research.

Little exists in the literature on the use of neuroaesthetics in the therapeutic applications of art and within the psychotherapeutic encounter, although this is an important and timely goal (Chatterjee, 2011). Hence, considering recent advances (Parada & Rossi, 2020), both theoretical and methodological frameworks are needed. As neuroaesthetics gains interest among broader scientific enquiry, the potential to expand its applications is pronounced while its definition is simultaneously challenged (Chatterjee & Vartanian, 2014; Di Dio & Gallese, 2009). Generating transdisciplinary research borne from a partnership of neuroaesthetics and art therapy might include a model for highly controlled perception–action (i.e., “low‐level”), ecologically valid naturalistic (i.e., “ecological level”), and real‐world translational studies (i.e., “applied level”). Such a partnership may help us understand how and why the arts are so helpful for the human condition in health and disease.

**FIGURE 1:**
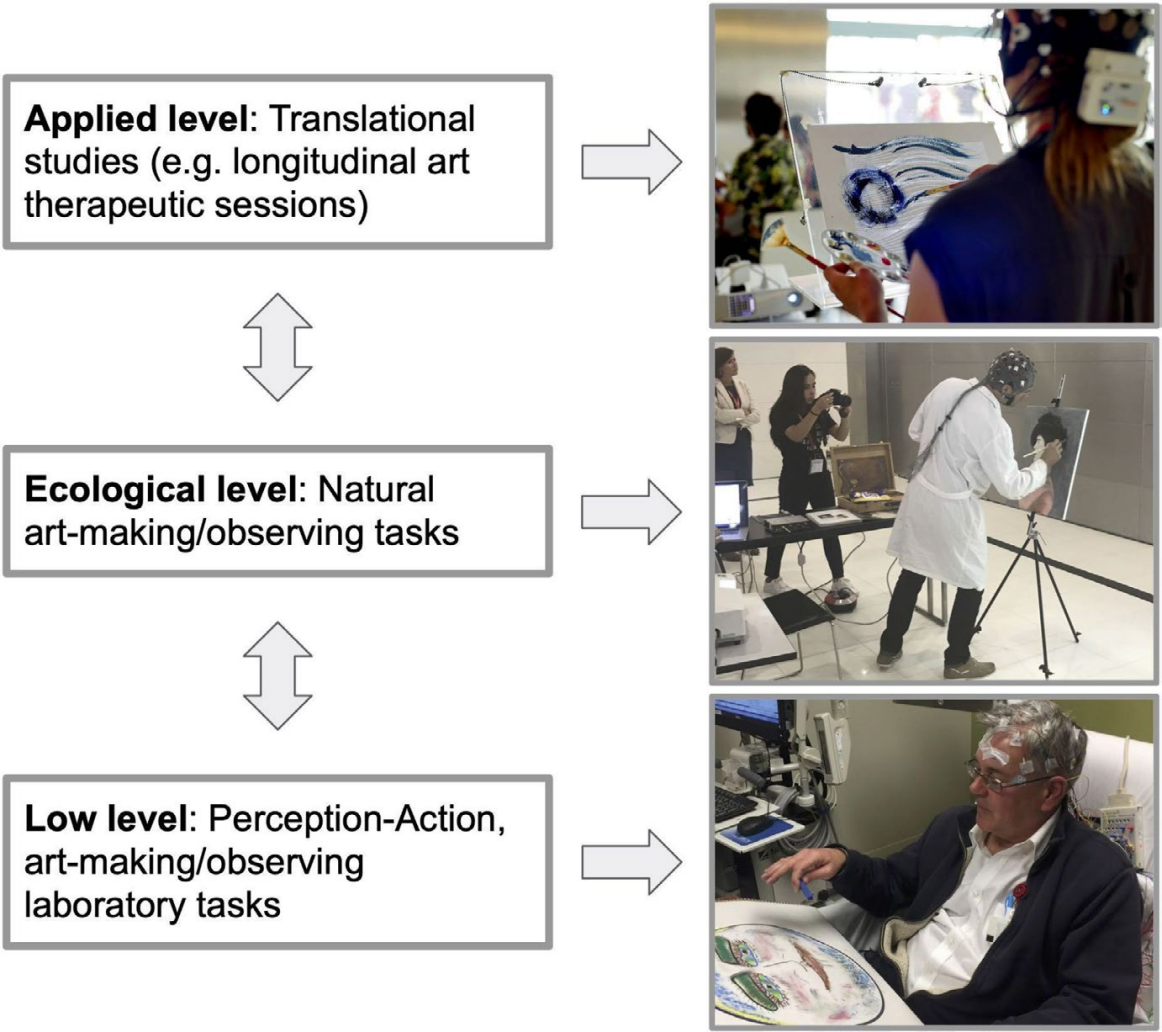
Three‐level Model for Neuroaesthetic Art Therapy research. Top Panel. Applied Level: This offers an example of what an art therapy session using MoBI technologies might look like for the client. Important to note that in most settings the therapist would also be present. In the image, hackathon participant, Stephanie Scott, wears a mobile EEG cap equipped with 32 dry electrodes. EEG activity is processed online and extracted band power values are used in a closed‐loop neurofeedback application. The background of a drawing canvas is updated live in response to, as well as a cause of her drawings (Photograph by Lukas Gehrke, Valencia, Spain, 2017. In Scott et al., 2019; and Scott & Gehrke, 2019). Ecological Level: An example of making art 'in the wild.'. MoBI facilitates the acquisition of data (Photograph by Juliet King, Valencia, Spain, 2017). Low Level: Capturing EEG data during an experiment using standard EEG at a more traditional setting such as a laboratory or a hospital (Photograph by Juliet King, Indianapolis, IN, 2017)

The three‐level model follows the scalable design logic suggested as a desirable gold standard in the MoBI and real‐world neuroscience literature (Matusz et al., 2019; Parada, 2018; Reiser et al., 2020; Shamay‐Tsoory & Mendelsohn, 2019). Figure 1 depicts the proposed three levels, bidirectional arrows show that any kind of work, at any level, can lead to work on any of the other levels (as suggested by Matusz et al., 2019; and Parada, 2018). The low level involves highly controlled perception–action laboratory experiments. The low‐level experiments can also be implemented in a hospital or clinic. These could be implemented through computer‐based and virtual reality settings, and/or the use of physical materials. Importantly, given that these are not ordinary perception studies, experimental designs must try to reach psychophysics rigor while carefully including first‐person experiences (Petitmengin & Lachaux, 2013). The main advantage of studies within this level is better signal‐to‐noise ratio, replicability, and control over variables, to name a few. The ecological level tries to replicate and/or extend low‐level results in a systematic way, focusing on the use of the MoBI framework within controlled natural and/or virtual reality settings. The MoBI framework has already implemented such a goal (e.g., Djebbara, Fich, Petrini, et al., 2019; Ladouce et al., 2019), and future neuroaesthetics/art therapy research should draw on these precedents.

Here, we refer to *ecological* following Brunswik (1943), where experimental designs should be “representative of the structure of the environment” (p. 261). Hence, the intermediate ecological level should try and focus into representing the different “ecologies,” and maximizing functional regularities belonging to art making/observing. Finally, the applied level points toward producing real‐world evidence in conditions that are simply not possible using more classical settings (Shamay‐Tsoory & Mendelsohn, 2019), such as therapeutic encounters (García & Di Paolo, 2018; Koole et al., 2020; Parada & Rossi, 2020). This level is closer to the idea of *Pasteur’s quadrant*, where the logic underlying knowledge production changes its focus (Tierney & Holley, 2008).

A current study example might be used to highlight how MoBI can serve to clarify meaningful changes in the cortical activation patterns of trauma survivors that differ from a normal population and show how these can be used to track and correlate with patient improvement in the setting of art psychotherapy. Investigating the therapeutic impact on brain dynamics offers MoBI as a new diagnostic and therapeutic tool that can help delineate intervention strategies based on neural correlates of artistic expression with different media and patient preference. This study would seek to determine the general characteristics of artistic expression using a range of materials within an art therapy protocol, and then explore how the brain/body substrates of artistic expression inform the cognitive, emotional, behavioral, and psychological conditions of trauma. MoBI could be used to identify the neural mechanisms associated with subjective perceptual experiences through the monitoring of EEG patterns and other visceral signals, such as HRV, while eye movement analysis could contribute to understanding the participants’ overt visual attention patterns in response to their own artwork and making processes. Measuring the sensory and motor properties of artistic materials could enhance our understanding of the biologically based subjective perceptual experiences that influence health and wellness (Park and Tallon‐Baudry, 2014). MoBI recordings of neurodynamics, visceral signals, and eye movements/fixations could then be associated (e.g., correlation and regression) with therapeutic outcome measures including participant reports. Ideally, this strategy would augment the predictive power for selection of optimal artistic media for individual patients. Thus, MoBI could help to ground neuroscience in clinical practice. This prospective controlled study would compare brain/body physiological dynamics in a traumatized population with normal controls to determine how outcome‐related activation patterns differ in post‐traumatic stress and determine how such changes improve in the setting of art therapy intervention.

The study could recruit patients who have experienced trauma for six art therapy sessions over an 8‐week timeframe. The study will include a series of questionnaires and self‐report measures to collect data on a variety of symptoms and experiences. Six art therapy sessions will be conducted while MoBI technology would record EEG data, HRV, and eye tracking. Data will be compared to cortical activation patterns assessed with a normal population and also with historical pilot data that demonstrated stationary EEG to be a meaningful tool for detection and quantification of cortical activation and processing in creative arts expression (King et al., 2017). This multi‐media art therapy protocol will be integrated into the work of “treatment as usual” and will enhance feasibility of subject recruitment and retention while maintaining the natural course of psychotherapy treatment for the participants. The purpose of this study is to investigate how brain signals and physiological measures change during engagement in artistic activity during the therapeutic encounter for patients with post‐traumatic stress. Such changes in cortical activation will provide insight into physiological processes in post‐traumatic stress and related disorders, serve as a surrogate or biomarker for quantifying severity of post‐traumatic stress and subsequent improvement, and offer a potential tool to predict those patients best suited for a specific therapeutic treatment regimen. It is anticipated that different frequencies of brain wave activity will be assessed pre‐ and post‐intervention and also during the art therapy process.

Studies have used EEG to explore brain wave patterns and functional connectivity in people diagnosed with post‐traumatic stress disorder (PTSD) and psychological trauma and found changes compared to healthy controls (Cook et al., 2009; Lobo et al., 2015; Shim et al., 2017). Based on these data, Gaskell (2018) provided a series of questions and suggested methodology to help understand the relationship between art making and access to traumatic memories as measured through EEG. Further studies have demonstrated that people with PTSD showed increased alpha and beta coherence over the central and temporal areas and increased functional connectivity in the parietal lobe (Cook et al., 2009; Imperatori et al., 2014). A systematic review of the literature on EEG correlates of the severity of post‐traumatic stress symptoms found that principle correlations remained significant and demonstrated a relative right‐hemispheric frontal and parietal activation that was associated with traumatic stress severity, and that there were significant correlations between global peak alpha frequency and overall symptom severity of re‐experiencing, avoidance, and hyperarousal (Lobo et al., 2015), and it was found that alpha measures are potential biomarkers for the severity of traumatic stress. Interestingly, visceral signals (i.e., gastric and heart dynamics) have been linked to alpha brain rhythms within the parietal‐occipital sulcus, right anterior insula, occipital lobe, and thalamocortical networks (Richter et al., 2017; Lechinger et al., 2015). It could be, thus, hypothesized that—given alpha rhythms have been associated with self‐regulation, relaxation, memory, visual processing, and creativity (Belkofer et al., 2014)—alpha waves could be an appropriate candidate to be measured as neurobehavioral markers for the characterization of pre‐, during, and post‐intervention neurodynamics in relation with self‐report scales showing that engagement in art therapy contributes to decreased symptomatology.

Hence, such a protocol seeks to record EEG prior, during, and after art therapy intervention to assess the impact of the intervention on brain signals. Further comparison to previous pilot data (King et al., 2017) illuminates the potential to evaluate the neural correlates of creative activity within the therapeutic encounter. Likewise, some specific aims for future studies could be: (a) to determine how patterns of brain/body activation differ from normal controls in patients with post‐traumatic stress and/or related disorders; (b) to determine if improvement in post‐traumatic stress symptoms with art therapy interventions is correlated with change and improvement in brain/body activation; (c) To determine if cortical activation differs with the use of specific art materials (i.e., media); and (d) To establish and clarify the efficacy of art therapy in specific populations (e.g., trauma and acquired brain lesion). Nevertheless, outstanding questions could be tackled in research programs, such as is *change* in measures of brain/body activation a useful surrogate or biomarker for clinical improvement? Does it offer a quantitative tool for studying conditions, such as PTSD, in response to therapy in investigational therapeutic trials? Do brain/body activation patterns predict which kinds of media are more likely to be successful in a particular patient?

In order to further interdisciplinary research, in addition to collecting a range of self‐report scales, baseline measures should be taken in the first and last therapeutic sessions. For example, resting‐state brain/body dynamics (e.g., eyes open/closed EEG/EKG recordings) could take place in each session prior to engaging in materials‐specific art therapy protocols. Pre‐ and post‐rote motor movement tasks (e.g., flipping a coin) and art assessment (e.g., mandala free drawing) would be taken pre‐ and post‐intervention. Another highly relevant topic includes data analysis protocols. These could consist of: (a) comparison of hypothesis‐driven brain/body dynamics from patients against controls, for example, alpha frequency power/phase and topography; and (b) compare pre‐ and post‐intervention measures. Patients will be assessed pre‐ and post‐intervention with standardized scales for trauma symptoms, trauma symptom severity, overall quality of life, and capacity to identify and describe emotions to establish the degree of objective benefit from specific interventions. Changes in these outcome measures will be compared with the changes seen in the pre‐ and post‐brain/body recordings to determine if the observed patterns change is in direct correlation with improvement or lack thereof in symptoms. This would establish whether the quantified activation measures as recorded will serve as a surrogate or biomarker for clinical improvement and, thus, add a quantitative measure to future outcome studies and clinical trials. In addition, these correlations will determine if there is a predictive value in the pattern of cortical activation seen pre‐therapeutic intervention and, thus, indicate and select patients for various types of specific interventions.

As further confirmed by the present special edition, measuring and understanding neurophysiological dynamics within ecological contexts is one of the most exciting recent scientific advances (Gramann et al., 2014; Ladouce et al., 2017; Makeig et al., 2009). However, although traditional cognitive science has been largely confined to the laboratory, the challenge to translation has primarily been because of technical limitations. Cognitive studies in small, not representative, and unnatural contexts have been recognized as a problem by experimental scientists throughout the 20th century (Bronfenbrenner, 1977; Brunswik, 1943; Neisser, 1976). Hence—even though just starting to match the theoretical demands of the 4E framework for the study of cognition—the recent advances in MoBI technology lend itself to integrate a neuroaesthetics and art therapy research program.

## 2 CONCLUDING REMARKS

As technological advances are moving rapidly, questions that have historically been avoided can now be approached. In particular, we refer to questions that explore complex phenomena related to human perception and behavior in therapeutic encounters. While traditional brain imaging has propelled our understanding of structures and anatomical changes in health and disease states, and systems of connectivity have been demonstrated with tracking brain activity in real time, MoBI brings these investigations to identifying brain dynamics *in the wild*.

Studies clarifying links among brain structure/functions, visceral states, and behavioral change through physiological markers are important in the development of research designs in therapeutics and are made more possible with non‐invasive MoBI technologies. MoBI can offer new insights into neurobiological mechanisms of change in psychotherapy that include artistic expression and dialogue within the therapeutic relationship. Researcher‐clinicians in the creative arts therapies can enhance the knowledge of the experimental scientist and offer practical methods for identifying behaviors, patterns of artistic self‐expression, clinical presentation, and interpersonal dynamics.

We think one of the highlights of MoBI’s first decade was the—mostly open source—ground‐breaking hardware and software solutions, allowing simultaneous acquisition of brain (e.g., EEG), behavior (e.g., eye tracker), and physiology (e.g., EKG) information in real time, during movement and/or in ecological environments (Gramann et al., 2014; Kothe, 2019; Makeig et al., 2009; Mullen et al., 2015; Ojeda et al., 2014). A clear goal for the next decade is to develop transdisciplinary research efforts integrating both basic and applied science (Parada & Rossi, 2020; Wolfe, 2016). Neuroaesthetics and art therapy encompassed by our three‐level model suggest a framework for transdisciplinary research efforts. Transdisciplinary research that includes scientists in the natural and social realms—along with medical and mental health professionals who use artistic expression to heal—illuminates the potential to generate novel hypotheses about the mind. Likewise, MoBI enables testing practical and ecologically valid applications in psychology, and psychotherapy brings opportunities such as (a) the possibility to identify and diagnose neuropsychiatric diseases in a non‐invasive way at a reduced cost, and (b) acquisition of multiple signals from participants in everyday situations, thereby expanding our range of research.

Art therapy is a form of psychotherapy, and its efficacy and effectiveness can be tested similarly to verbal psychotherapy with a standardization of identified variables. Introducing art therapy research to neuroscience as a means to explore the underpinnings of sensory, motor, and cognitive processes involved can help to conceptualize and further hypothesize the underlying mechanisms of psychological change throughout treatment (Beans, 2019). Scientific evidence expands the reach of art therapy, tying the components together in a systematic theory‐driven way, yet providing enough space for the ambiguity of human experience that transcends any correlational design. Physiological data provide insights and are increasingly reliable (Seeber et al., 2019) but do not provide a functional/behavioral explanation for the essence of experience (Krakauer et al., 2017). During the present work, only one mixed‐methods EEG study was found (Tilley et al., 2017) that utilized participant responses along with physiological data. Using physiological data with behavioral measures and self‐report scales has yet to be truly explored as a first step (Varela, 1996), but is an important methodology to consider when translating scientific findings to clinical health populations. We provided an example for the use of MoBI to clarify meaningful changes in the cortical activation patterns of trauma survivors that differ from a normal population and show how these can be used to track and correlate with patient improvement in the setting of art psychotherapy. Investigating the therapeutic impact on brain dynamics offers MoBI as a new diagnostic and therapeutic tool that can help delineate intervention strategies based on neural correlates of artistic expression with different media and patient preference. Art therapy encounters are an example of an ecological, semi‐structured setting in which the nature of dynamic change is what is most important. MoBI is a promising technology to move forward in linking ideas from neuroaesthetics to research in art therapy.

In sum, we are at a key moment to advance as a research community by solving these challenges and moving toward a cognitive science with greater ecological validity; one that does not confine the study of cognitive processes to understanding intracerebral phenomena to the laboratory, but instead takes full account of the body and environment in which cognition arises. These efforts will be served when translated to clinical health populations in the development of best practices for treatment and research.

## CONFLICT OF INTEREST

The authors declare that the research was conducted in the absence of any commercial or financial relationships that could be construed as a potential conflict of interest.

## AUTHOR CONTRIBUTIONS

JLK and FJP conceptualized, wrote and edited the present manuscript for publication.

### PEER REVIEW

The peer review history for this article is available at https://publons.com/publon/10.1111/ejn.15313.

## Data Availability

The present work is a theoretical piece which includes no shareable original data. Nevertheless, any included figures/tables are available upon request to the corresponding author Juliet L. King.

## Footnotes

^1^
In contrast to traditional art therapy research, the search for mechanisms has a long tradition in neural sciences (Bechtel, 2012; Craver, 2007; Rojas‐Líbano & Parada, 2019).

^2^
That is, Cognition is an Embodied, Extended, and Embedded phenomenon, better studied from an Enactive perspective
